# Crystal structure of the anti-CRISPR repressor Aca2

**DOI:** 10.1016/j.jsb.2021.107752

**Published:** 2021-09

**Authors:** Ben Usher, Nils Birkholz, Izaak N. Beck, Robert D. Fagerlund, Simon A. Jackson, Peter C. Fineran, Tim R. Blower

**Affiliations:** a Department of Biosciences, Durham University, Stockton Road, Durham DH1 3LE, UK; b Department of Microbiology and Immunology, University of Otago, PO Box 56, Dunedin 9054, New Zealand; c Genetics Otago, University of Otago, PO Box 56, Dunedin 9054, New Zealand; d Bio-Protection Research Centre, University of Otago, PO Box 56, Dunedin 9054, New Zealand

**Keywords:** CRISPR, X-ray crystallography, Anti-CRISPR associated, Transcriptional regulator, Aca2

## Abstract

Bacteria use adaptive CRISPR-Cas immune mechanisms to protect from invasion by bacteriophages and other mobile genetic elements. In response, bacteriophages and mobile genetic elements have co-evolved anti-CRISPR proteins to inhibit the bacterial defense. We and others have previously shown that anti-CRISPR associated (Aca) proteins can regulate this anti-CRISPR counter-attack. Here, we report the first structure of an Aca protein, the Aca2 DNA-binding transcriptional autorepressor from *Pectobacterium carotovorum* bacteriophage ZF40, determined to 1.34 Å. Aca2 presents a conserved N-terminal helix-turn-helix DNA-binding domain and a previously uncharacterized C-terminal dimerization domain. Dimerization positions the Aca2 recognition helices for insertion into the major grooves of target DNA, supporting its role in regulating anti-CRISPRs. Furthermore, database comparisons identified uncharacterized Aca2 structural homologs in pathogenic bacteria, suggesting that Aca2 represents the first characterized member of a more widespread family of transcriptional regulators.

## Highlights

• The crystal structure of the anti-CRISPR repressor Aca2 has been solved to 1.34 Å.
• Aca2 contains a new dimerization domain for HTH transcriptional regulators.
• Aca2-like regulators are found encoded in diverse biological contexts.

## Graphical abstract

**Figure:**
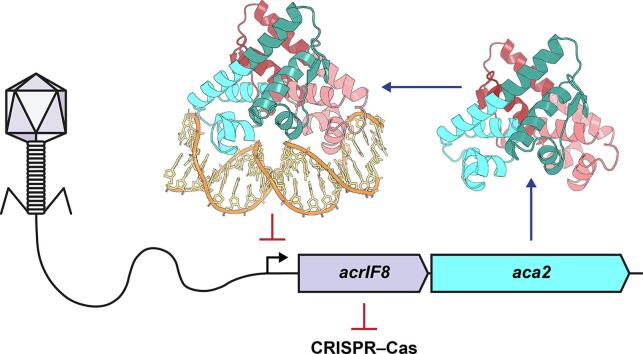

## 1 Introduction

Bacteria are under constant threat of invasion by bacteriophages (phages) and other mobile genetic elements (MGEs). Among the many protection strategies employed against these invaders, the highly diverse CRISPR-Cas systems stand out as the only known adaptive immune systems in bacteria (Hampton et al., 2020). In response, phages and MGEs have evolved a large array of anti-CRISPR (Acr) proteins which can inhibit CRISPR-Cas defense through various means (Malone et al., 2021, Wiegand et al., 2020). With different Cas proteins, such as Cas9, being utilized as tools in bioengineering, Acr proteins offer a way to make these tools more controllable and may substantially facilitate their application (Marino et al., 2020).

**Fig. 1:**
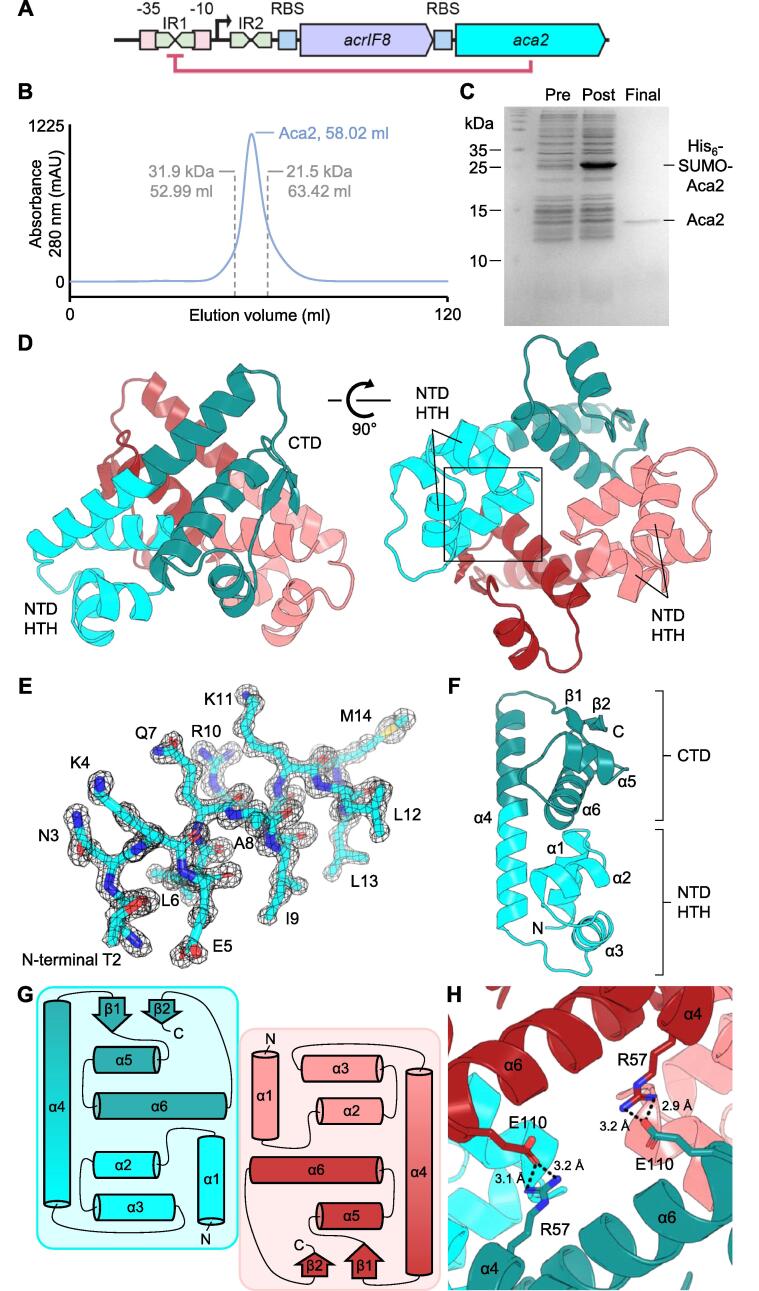
Structure of Aca2. (A) Architecture of the *acrIF8–aca2* locus from *P. carotovorum* phage ZF40 (not to scale). Promoter elements (−10 and −35 regions), inverted repeats (IR1 and IR2), transcription start site (arrow) and ribosome-binding sites (RBS) are indicated. Aca2 binding to IR1 and repressing transcription is shown in red. (B) Elution volume of untagged Aca2 during size-exclusion chromatography (SEC) shows it is a dimer in solution. (C) SDS-PAGE of pre-induction (Pre), post-induction (Post), and cleaved, purified Aca2 protein (Final). (D) Cartoon overview of the Aca2 dimer, with one protomer shown as cyan (NTD) and teal (CTD), and the other protomer shown as pink (NTD) and red (CTD). Two orthogonal views are shown, rotated by 90°. NTD HTH – N-Terminal Domain Helix-Turn-Helix. CTD – C-Terminal Domain. (E) Boxed region of (D), containing helix α1 as sticks, shown with a 2Fo-Fc electron density map contoured to 2σ. (F) Single protomer of Aca2 with secondary structures and domains labelled. (G) Topology of the Aca2 dimer. (H) Close-up top view of the Aca2 dimer, rotated down by 90° from (D, left panel) showing salt bridges between protomers. Distances shown are in angstroms.

Many anti-CRISPR genes form an operon with genes encoding anti-CRISPR-associated (Aca) proteins, ten families of which have been identified (Bondy-Denomy et al., 2013, He et al., 2018, León et al., 2021, Marino et al., 2018, Pawluk et al., 2016a, Pawluk et al., 2016b, Pinilla-Redondo et al., 2020, Yin et al., 2019). For example, *Pseudomonas aeruginosa* phage JBD30 contains an *acrIF1–aca1* operon and *Pectobacterium carotovorum* phage ZF40 contains an *acrIF8–aca2* operon (Fig. 1A). We and others recently showed that Aca1 and Aca2, as well as Aca3 encoded in an *acrIIC3–aca3* operon, serve as repressors of their respective promoters (Birkholz et al., 2019, Stanley et al., 2019). These findings and the pervasive presence of helix-turn-helix (HTH) domains in all known Aca proteins suggest that Aca proteins generally function to repress, or at least to regulate, anti-CRISPR production. In some cases, the anti-CRISPR itself contains an HTH domain for autoregulation (Osuna et al., 2020). Interestingly, bacteria may use their own Aca-like regulators to inhibit anti-CRISPR deployment by phages, thereby maintaining CRISPR-Cas defense (Osuna et al., 2020, Stanley et al., 2019).

Aca1 and Aca2 bind to inverted repeats (IRs) that overlap with the −10 and −35 elements of their respective promoters, suggesting that transcriptional repression occurs through blocking of RNA polymerase recruitment (Birkholz et al., 2019, Stanley et al., 2019). The *acrIF8–aca2* promoter contains two similar IR pairs and IR1 was shown to be bound tightly by Aca2 (Fig. 1A). We demonstrated this interaction involves DNA bending, thus providing a first insight into the topological changes involved in anti-CRISPR regulation (Birkholz et al., 2019). However, information on the structural basis of Aca-mediated repression is still missing. In this study, we determined the crystal structure of the anti-CRISPR-associated protein Aca2 from *P. carotovorum* phage ZF40 to better understand its role as a transcriptional regulator.

## 2 Materials and methods

### 2.1 Aca2 cloning

The *aca2* gene was amplified from pPF1575 (Birkholz et al., 2019) with primers TRB1765 (5′-CAACAGCAGACGGGAGGTACAAACAAAGAACTTCAGGC-3′) and TRB1766 (5′-GCGAGAACCAAGGAAAGGTTATTATTAGATTAAATCCGCGTGACC-3′), then cloned into pSAT1-LIC (Cai et al., 2020) via ligation-independent cloning (LIC) (Aslanidis and de Jong, 1990), to produce pTRB627. The pSAT1-LIC plasmid features a LIC site that fuses an N-terminal His_6_-SUMO tag to the target protein.

### 2.2 Recombinant protein expression

Aca2 was expressed in *E. coli* BL21(λDE3)Δ*slyD* (Cai et al., 2020) transformed with pTRB627. Overnight cultures were re-seeded 1:100 into 2 L baffled flasks containing 1 L 2×YT. Cells were grown at 160 rpm, 37 °C, until an OD_600_ of 0.3 was reached and then at 25 °C until OD_600_ 0.6. Expression was induced by the addition of IPTG (1 mM), then cells were left to grow overnight at 16 °C, with shaking at 160 rpm.

### 2.3 Recombinant protein purification

Following overnight expression, bacteria were harvested by centrifugation at 4,200 *g*, 4 °C, and the pellets were resuspended in buffer A [20 mM Tris-HCl (pH 7.9), 500 mM NaCl, 5 mM imidazole, and 10% glycerol]. Cells were lysed by sonication at 40 kpsi and then centrifuged at 45,000 *g*, 4 °C. The clarified lysate was then passed down a HisTrap HP column (Cytiva) using a peristaltic pump. The resin-bound protein was first washed for 10 column volumes with buffer A, followed by 10 column volumes of buffer B [20 mM Tris-HCl (pH 7.9), 100 mM NaCl, 35 mM imidazole, and 10% glycerol] and then eluted directly onto a HiTrap Q HP column (Cytiva) with buffer C [20 mM Tris-HCl (pH 7.9), 100 mM NaCl, 250 mM imidazole, and 10% glycerol]. The Q HP column was washed briefly with 5 column volumes of buffer D [20 mM Tris-HCl (pH 7.9), 100 mM NaCl, 5 mM imidazole, and 10% glycerol], and then transferred to an Äkta Pure (Cytiva). Proteins were separated using an elution gradient from 100% buffer D to 40% buffer E [20 mM Tris-HCl (pH 7.9), 1 M NaCl, and 10% glycerol]. Fractions corresponding to the chromatogram protein peak were pooled and incubated overnight at 4 °C with hSENP2 SUMO protease to cleave the N-terminus His_6_-SUMO tag from recombinant Aca2. The next day, the sample was passed through a second HisTrap HP column via a peristaltic pump, then washed for 2 column volumes with buffer A. The flow-through and wash fractions containing untagged Aca2 were collected and concentrated, then loaded onto a HiPrep 16/60 Sephacryl S-200 size exclusion column (Cytiva) connected to an Äkta Pure, in buffer S [50 mM Tris-HCl (pH 7.9), 500 mM KCl, and 10% glycerol]. Fractions corresponding to the chromatogram peak were analyzed by SDS-PAGE, with optimal fractions then pooled and dialyzed overnight at 4 °C into buffer X [20 mM Tris-HCl (pH 7.9), 150 mM NaCl, and 2.5 mM dithiothreitol (DTT)] for crystallography. Crystallography samples were concentrated, quantified, and stored on ice, then either used immediately or flash-frozen in liquid N_2_ for storage at −80 °C.

### 2.4 Protein crystallization

Aca2 was concentrated to 12 mg ml^−1^ in buffer X. Crystallization screens were performed using a Mosquito Xtal3 robot (STP Labtech) to set 200:100 nl and 100:100 nl protein:condition sitting drops. Initial crystals formed 6 days after incubation at 294 K and were left to grow until day 29. Aca2 was observed to form small needle-like crystals in condition B6 of Structure 1 + 2 Eco (Molecular Dimensions) [0.2 M sodium acetate trihydrate, 100 mM MES (pH 6.5), and 30% w/v PEG 8000], at a final protein concentration of 8 mg ml^−1^. The Aca2 crystals were harvested directly from the screen. To harvest, 20 μl of the condition reservoir was added to 20 μl of cryo buffer [25 mM Tris-HCl (pH 7.9), 187.5 mM NaCl, 3.125 mM DTT, and 80% glycerol] and mixed quickly by vortexing. An equal volume of this mixture was then added directly to the crystal drop, and the Aca2 crystal was immediately extracted using a nylon loop and flash-frozen in liquid N_2_.

### 2.5 Data collection and structure determination

Diffraction data were recorded at 100 K on beamline I04 at Diamond Light Source. A single 360° dataset was collected for Aca2. Diffraction data were processed with XDS (Kabsch, 2010), and then AIMLESS in CCP4 (Winn et al., 2011) was used to corroborate the space group. The crystal structure of Aca2 was solved *ab initio* using ARCIMBOLDO (Rodríguez et al., 2009), with initial model-building then performed using Buccaneer (Cowtan, 2006) in CCP4 (Winn et al., 2011). Data processing then moved to PHENIX (Adams et al., 2010) and COOT (Emsley and Cowtan, 2004), where the model was iteratively refined and built, respectively. The quality of the final model was assessed using COOT and the wwPDB validation server (Gore et al., 2012). Structural figures were generated using PyMol (Schrödinger). RMSD values were calculated with the Super command in PyMol, using all atoms and then rejecting outlier pairs.

## 3 Results

### 3.1 Overall structure of Aca2

Aca2 was expressed and purified as described (Materials and Methods). The elution volume during the final size exclusion chromatography run indicated that the 13.7 kDa Aca2 protein forms a dimer in solution (Fig. 1B), corroborating what was observed with our previous constructs (Birkholz et al., 2019). This final Aca2 product was judged sufficiently pure for crystallization by SDS-PAGE (Fig. 1C). Using this sample, we determined the crystal structure of Aca2 to 1.34 Å (Fig. 1D) and refined the structure to an R-factor of 0.1476 and an R-free of 0.1761 (Table 1).

**Table 1:** Data collection and refinement statistics for Aca2.

PDB ID code	75BJ
*Data Collection*	
Beamline	Diamond I04
Wavelength (Å)	0.9795
Resolution range (Å)^a^	38.19–1.34 (1.388–1.34)
Space group	*P*2_1_
Unit cell, *a b c* (Å); α β γ (°)	39.791, 67.103, 42.240; 90, 106.331, 90
Total reflections^a^	646,247 (63250)
Unique reflections^a^	47,804 (4740)
Multiplicity^a^	13.5 (13.3)
Completeness (%)^a^	99.98 (99.96)
Mean *I*/σ(*I*)^a^	10.28 (1.18)
*R_merge_*^a^, ^b^	0.1588 (1.688)
CC_1/2_^a^	0.999 (0.663)
*Refinement*	
*R_work_*^c^	0.1476 (0.2103)
*R_free_*^c^	0.1761 (0.2509)
Number of non-hydrogen atoms	2125
macromolecules	1854
ligands	6
solvent	265
Protein residues	230
RMS (bonds, Å)	0.009
RMS (angles, °)	1.07
Ramachandran favored (%)	99.12
Ramachandran allowed (%)	0.88
Ramachandran outliers (%)	0.00
Rotamer outliers (%)	0.00
Clashscore	0.82
Average B-factor	19.31
macromolecules	17.48
ligands	24.20
solvent	32.04

a Statistics for the highest resolution shell are shown in parentheses.

b *R*_merge_ = Σ_h_Σ_i_|*I_h_,_i_-I_h_*|/Σ_h_Σ_i_*I_h_*,*_i_*, where *I_h_* is the mean intensity of the *i* observations of symmetry related reflections of *h*.

c *R*_work_/*R*_free_ = Σ|*F_obs_*-*F_calc_*|/Σ*F_obs_*, where *F_calc_* is the calculated protein structure factor from the atomic model (*R*_free_ was calculated with 5% of the reflections selected).

Previous work identified a putative N-terminal domain (NTD) containing an HTH motif required for DNA binding (Birkholz et al., 2019). The Aca2 dimer structure shows each protomer stacked against one another in opposition, like the letter X, such that the HTH motifs are aligned along the “base” of the dimer (Fig. 1D). The obtained data (Table 1) allowed all amino acids within an Aca2 dimer to be modelled (Fig. 1D), and an example section of the Aca2 2Fo-Fc electron density map is shown for the first alpha-helical region, beginning with the N-terminal amino acid T2 (Fig. 1E). Examining a single protomer shows that the proposed NTD HTH and relative C-terminal domain (CTD) are in fact small clusters of secondary structure elements abutting and joined by a longer backbone α-helix, α4, such that the protomer forms a single globular protein (Fig. 1F). All Aca2 residues (116 amino acids in total) are resolved in the structure except the initial methionine, which was not included in the construct. Aca2 is comprised of 6 α-helices; α1 (amino acid (aa) positions 2–13), α2 (aa 16–24), α3 (aa 28–38), α4 (aa 43–70), α5 (aa 81–89) and α6 (aa 93–110). A short β-strand, β1 (aa 74–78), is encoded between α4 and α5, and forms a very short two-stranded parallel β-sheet with β2 (aa 114–116) (Fig. 1F). An HTH motif contains an α-helix for positioning, and an α-helix for DNA recognition, linked by a short turn. In Aca2, α2 will help position α3 for DNA recognition (Fig. 1F). This is further supported by a previous mutagenesis study that showed R30 was necessary for promoter autoregulation by Aca2, and R30 is found on α3 (Birkholz et al., 2019). Whilst the NTD provides the HTH motif for DNA-binding, the CTD stabilizes the positioning of the NTD from the other protomer by stacking α6 against the other protomer α1, thereby aiding dimerization. This interaction forms the bulk of the dimer interface and can be seen both in the provided views (Fig. 1D), as well as schematically within the topology diagram (Fig. 1G). The Aca2 protomer-protomer interface was analyzed using PDBsum (Laskowski et al., 2018), which calculated a buried surface area of 1213 Å^2^. This is supported by two salt bridges formed between R57 within α4 of protomer A and E110 within α6 of protomer B, and vice versa (Fig. 1H). Hydrogen bonds were calculated by PDBsum as forming between F78 of protomer A and K4 of protomer B (and again vice versa). The rest of the interface is proposed to form through van der Waals interactions. This solved structure shows a stable Aca2 dimer forming a single globular unit with the HTH domains positioned to recognise DNA sequences.

### 3.2 Analysis of the Aca2 dimer

**Fig. 2:**
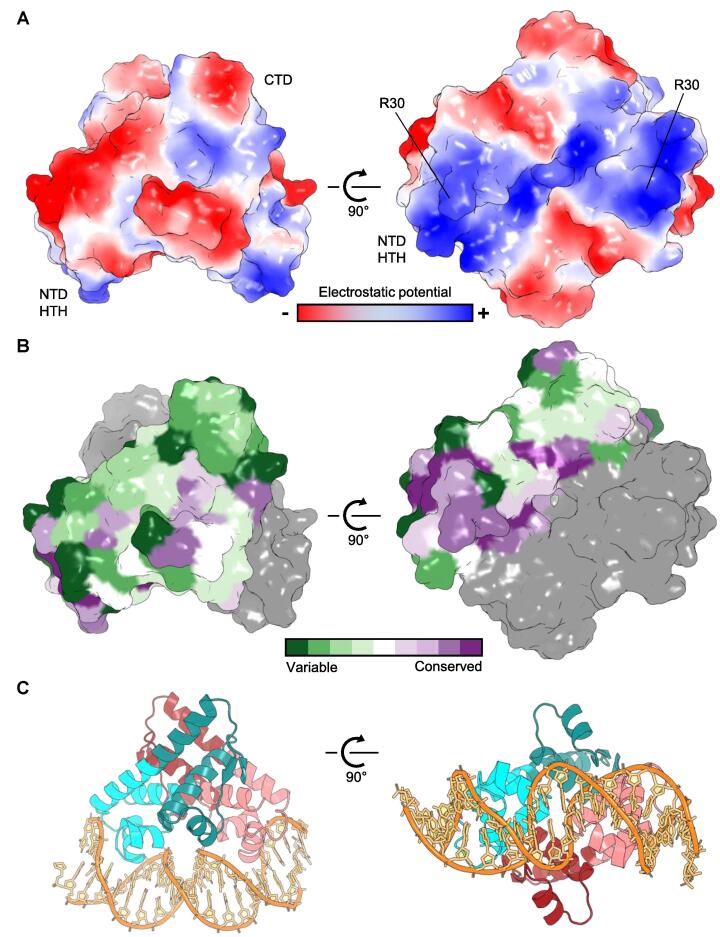
Analysis of Aca2 dimers. (A) Electrostatic surface potential shows electropositivity (blue) in the NTD HTH domains of Aca2 (left panel). There is an electropositive groove between the two HTH domains and respective key DNA-binding residues from each protomer, R30, are positioned ~ 30 Å apart (right panel). (B) Conservation plots on one Aca2 protomer (colored green to purple as per scale), shown in dimer form (second protomer in gray). (C) Aca2 dimer modelled in complex with 20 bp IR1 dsDNA.

Next, we examined the surface properties of the Aca2 dimer based on both electrostatic potential (Fig. 2A), and residue conservation (Fig. 2B). Whilst the upper CTD surface of the dimer contains mixed patches of both electropositive and electronegative potential, the NTD HTH motif is clearly electropositive and primed for DNA binding (Fig. 2A, left). When rotated upwards 90° to visualize the “underside” of the Aca2 dimer, there is a clear groove of electropositivity across the entire underside that spans the ~ 30 Å separating the R30 residues and indicates the likely direction for DNA binding (Fig. 2A, right).

ConSurf (Ashkenazy et al., 2016) was used to select sequences homologous to Aca2 (Supplementary Material Table 1), perform a multiple sequence alignment (Supplementary Material Table 2), and then calculate residue conservation from these multiple alignments. This conservation output was then mapped onto the Aca2 surface (Fig. 2B). Interestingly, conservation showed a similar distribution to the electrostatic potential, with greatest conservation in the areas of the HTH and proposed DNA-binding groove, whilst other sections of the α4 backbone helix and CTD were poorly conserved (Fig. 2B).

Previous data have shown that Aca2 autoregulates expression through both DNA binding and bending (Birkholz et al., 2019). Homology modelling via PHYRE 2.0 (Kelley et al., 2015) indicated potential structural homology between DNA-bound MqsA (PDB 3O9X) and Aca2, due to the presence of HTH motifs on both proteins (Birkholz et al., 2019). Having now obtained the Aca2 structure, alignment with MqsA through the HTH domains allowed us to propose a model for Aca2-DNA binding (Fig. 2C). This modelled DNA contains the 20 bp IR1 region of the Aca2 promoter (Fig. 1A) and the two recognition helices can be seen inserting into the major grooves. This also demonstrates how the observed DNA bending might be facilitated by complementary surfaces of Aca2, to allow insertion of the recognition helices (Fig. 2C).

### 3.3 Structural comparisons of Aca2

The DALI server (Holm and Sander, 1993) was used to search the PDB for structural homologs of Aca2 (Supplementary Material Table 3). The two highest scoring hits were for YdiL from *Salmonella enterica* subsp. *enterica* serovar Typhimurium LT2 (gene *ydiL* aka *STM1362*, PDB 1S4K), and SO3848 from *Shewanella oneidensis* MR-1 (gene *so3848*, PDB 2OX6). YdiL and SO3848 scored Z-scores of 16.6 and 11.9, respectively, and were the only hits that aligned with both domains of Aca2. The bacteria encoding *ydiL* and *so3848* are both γ-proteobacterial pathogens (Heidelberg et al., 2002, McClelland et al., 2001), as is *P. carotovorum*, the host for prophage ZF40 from which Aca2 is derived (Tovkach, 2002). Both the YdiL and the SO3848 structures were produced and deposited by the Midwest Center for Structural Genomics, and both proteins have no known biological function.

**Fig. 3:**
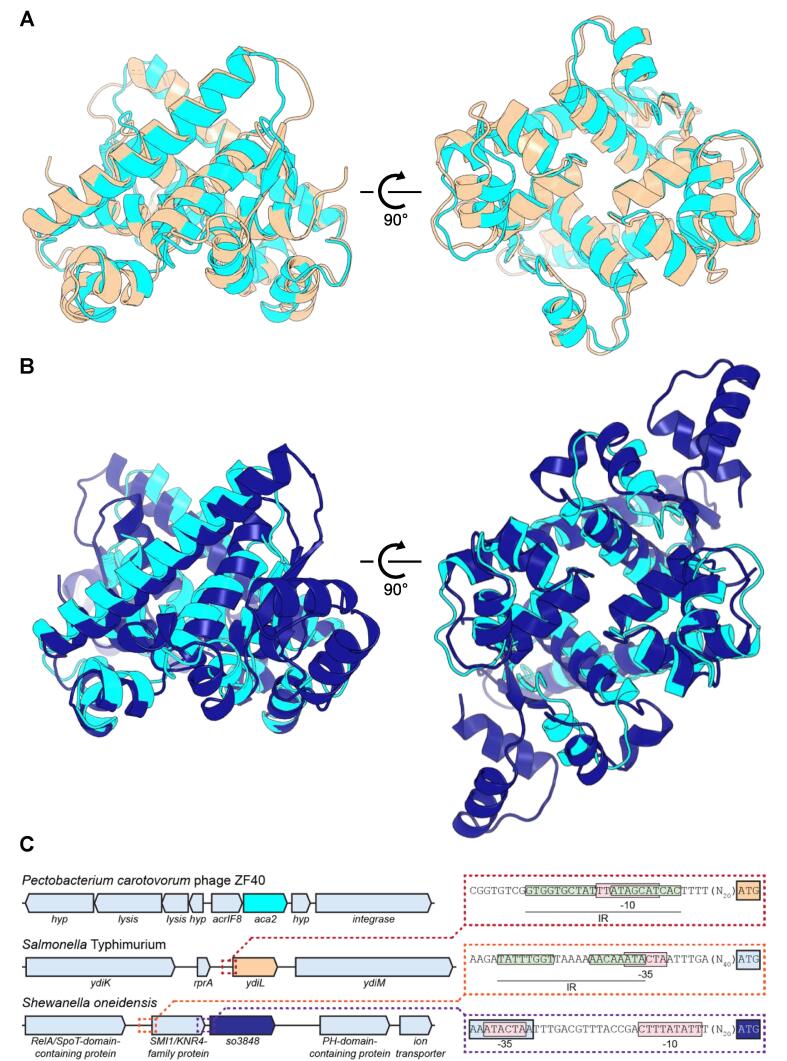
Structural homologs of Aca2. (A) Superposition of Aca2 dimer (cyan) with YdiL dimer (pale orange, PDB 1S4K). (B) Superposition of Aca2 dimer (cyan) with SO3848 dimer (dark blue, PDB 2OX6). (C) Genomic context of Aca2 structural homologs. *hyp*: gene encoding hypothetical protein. Promoter regions of interest are enlarged on the right; pink boxes indicate promoter elements (-10 and −35 regions), green boxes indicate inverted repeats; coding regions are indicated in the same color as in the overview on the left.

Aca2 comprises 116 amino acids, YdiL is 119 amino acids and SO3848 is 166 amino acids. Using EMBOSS Stretcher (Madeira et al., 2019), Aca2 and YdiL share sequence identity of 31.5%, Aca2 and SO3848 share sequence identity of 25.8%, and YdiL and SO3848 share sequence identity of 22.0%, which suggests they are all poorly related to one another at the sequence level. Despite poor sequence similarity, structure-based superposition of Aca2 and YdiL produced an RMSD of 1.8 Å, between 1420 atoms (Fig. 3A). This superposition shows that Aca2 and YdiL are highly similar at the structural level. In contrast, the structure-based superposition of Aca2 and SO3848 is relatively worse, with an RMSD of 3.6 Å, between 1292 atoms (Fig. 3B). Nevertheless, Aca2 and the core regions of SO3848 overlay well (Fig. 3B, left), with variations in secondary structure wherein the equivalent β1 in SO3848 is longer, SO3848 has an additional α-helix between the equivalents of α5 and α6, and β2 is also longer (again forming a parallel β-sheet with β1). SO3848 also has a unique extension to the CTD formed by two additional α-helices that can clearly be seen as additional decorations to the globular core (Fig. 3B, right). These alignments suggest that the biological function of both YdiL and SO3848 is to act as DNA-binding proteins and potential transcriptional regulators, perhaps autorepressors.

We examined the genomic contexts of both YdiL and SO3848 for further clues as to their function (Fig. 3C). Based on genome-wide expression profiling, *ydiL* was not expressed in any of 22 conditions tested in *Salmonella* (Colgan et al., 2016) and is not essential, as determined by TraDIS (Canals et al., 2019). Furthermore, *ydiL* is not part of a prophage (McClelland et al., 2001) or a genomic island, based on an IslandViewer analysis (Bertelli et al., 2017), suggesting the gene is part of the *S.* Typhimurium core genome. Assuming that YdiL is a regulatory protein, it is therefore possible that it binds to sites at distant genomic locations, especially considering its apparent stand-alone character (Fig. 3C) – a stark contrast to the *acrIF8–aca2* operon found in *P. carotovorum* phage ZF40, or *aca* genes in general. However, we also identified several inverted repeats in the vicinity of *ydiL*, including an IR overlapping with the −10 site that might mediate autorepression (Fig. 3C, red inset).

*S. oneidensis* SO3848 is encoded downstream of a gene encoding a predicted SMI1/KNR4-family protein (Fig. 3C), upstream of which we identified an IR overlapping the −35 site (Fig. 3C, orange inset). Bacterial homologs of the SMI1/KNR4 family have been implicated in contact-dependent inhibition systems (Zhang et al., 2011). Given the context of an Aca2-like regulator, it is possible that this gene has evolved to fulfil an alternative function as an anti-CRISPR; however, similar to *ydiL*, *so3848* does not appear to be part of a genomic island or prophage (as determined using IslandViewer (Bertelli et al., 2017), PHASTER (Arndt et al., 2016) and Prophage Hunter (Song et al., 2019)). From sequence analysis alone it is unclear whether *so3848* and its upstream gene form an operon; despite their close proximity, *so3848* appears to have its own promoter with BPROM-predicted −10 and −35 sites (Solovyev and Salamov, 2011) (Fig. 3C, purple inset). Global profiling showed that *so3848* is expressed in *S. oneidensis* (Kolker et al., 2005) and its expression level is affected by different terminal electron acceptors (Beliaev et al., 2005). The disparate genomic settings of the three Aca2 structural homologs (Fig. 3C) suggest diverse implementation of a conserved DNA-binding strategy.

## 4 Discussion

In this study we have determined the crystal structure of the Aca2 anti-CRISPR-associated transcriptional autorepressor. The obtained structure supports the earlier biological data on DNA binding and bending, with conserved residues lining the electropositive DNA-binding surfaces. We previously proposed that upon phage infection, anti-CRISPR expression initiates strongly and Aca2 will switch off anti-CRISPR production once the host defence has been shut down, potentially to reduce toxic side-effects of AcrIF8 (Birkholz et al., 2019). Should the phage enter lysogeny, Aca2 autoregulation would ensure AcrIF8 levels are suppressed, enabling the host to maintain some CRISPR–Cas activity and ensure protection from secondary infections.

Whilst the Aca2 NTD HTH is highly conserved, the CTD used for dimerization has not been previously characterized, suggesting Aca2 represents a new family of transcriptional regulators. Indeed, Aca2 stands out among the known Aca family members in terms of its larger size (Bondy-Denomy et al., 2018), with other, smaller Aca proteins likely consisting of only one domain (including the HTH motif) and therefore using other dimerization mechanisms. Two structural homologs of Aca2, both from bacterial pathogens, were identified in databases, although both were uncharacterized outputs from structural genomics efforts. Together, the three homologs appear in various genomic contexts, suggesting that the Aca2 family might be more extensively widespread, and that this structural scaffold may be involved in regulating a large range of biological processes. Further work will be needed to fully examine a DNA-bound structure and investigate more diverse members of this nascent family.

## 5 Accession number

The crystal structure of Aca2 has been deposited in the Protein Data Bank under accession number 7B5J.

## CRediT authorship contribution statement

**Ben Usher:** Investigation, Visualization, Writing - original draft. **Nils Birkholz:** Investigation, Visualization, Writing - original draft. **Izaak N. Beck:** Investigation, Visualization, Writing - original draft. **Robert D. Fagerlund:** Supervision, Writing - review & editing. **Simon A. Jackson:** Supervision, Writing - review & editing. **Peter C. Fineran:** Conceptualization, Funding acquisition, Supervision, Writing - original draft. **Tim R. Blower:** Conceptualization, Funding acquisition, Supervision, Investigation, Visualization, Writing - original draft.

## Declaration of Competing Interest

The authors declare that they have no known competing financial interests or personal relationships that could have appeared to influence the work reported in this paper.
